# Morphological Measurement and Clinical Significance of Abnormal Development of Distal Femur with Hemophilia Knee Arthritis: A Consideration on the Renewal of Total Knee Prosthesis

**DOI:** 10.1111/os.14170

**Published:** 2024-08-06

**Authors:** Ru Feng, Houlong Ye, Wang Fang, Chun Zhang, Renfei Qi, Juehua Jing, Yunfeng Yao

**Affiliations:** ^1^ Department of Orthopedics An'kang Central Hospital Ankang China; ^2^ Department of Orthopedics An'kang Orthopaedics Hospital Ankang China; ^3^ Department of Orthopedics, The Second People's Hospital of Hefei Hefei Hospital Affiliated to Anhui Medical University Hefei China; ^4^ Department of Orthopedics The Second Affiliated Hospital of Anhui Medical University Hefei China; ^5^ Institute of Orthopedics, Research Center for Translational Medicine The Second Affiliated Hospital of Anhui Medical University Hefei China

**Keywords:** Distal Femur, Hemophilic Arthritis, Matching Degree, Morphological Malformation, Osteotomy Surface

## Abstract

**Objective:**

The knee joint of hemophiliacs may face the result of local morphological changes due to long‐term irritation of synovitis. This study aims to elucidate the morphological characteristics of distal femur in hemophilic arthritis (HA) and compare the compatibility of three types of prostheses with the anteroposterior (AP) and mediolateral (ML) dimensions of the femoral osteotomy surface.

**Methods:**

This study retrospectively and randomly selected 50 patients with HA registered for treatment at our hospital from June 2016 to August 2022 as the study subjects, with an equal number of male osteoarthritis (OA) patients and healthy male individuals set as the control group. This study used medical digitalization software to simulate osteotomies on the distal femur during total knee arthroplasties (TKA) for 50 patients with HA, OA patients, and the healthy population, respectively, and measure the morphological parameters to compare with three commonly used femoral components of TKA in clinical practice. The differences between the femur resection of anteroposterior and mediolateral (FRAP, FRML) osteotomy surface and the prosthesis's BOX‐AP/ML were compared in three prostheses. One‐way ANOVA and multiple Kruskal–Wallis H test were used for the normal or non‐normal distribution data, and pairwise comparisons between groups were conducted using the Bonferroni method, and the linear correlation analysis was utilized to assess the relationship between section femoral morphological data and prosthesis parameters.

**Result:**

In HA patients, the morphological characteristics of the distal femur were shown as shorter than femur AP (FAP), medial and lateral condyle anterior–posterior dimension (FMCAP, FLCAP), notch width (NW), posterolateral condyle height (PLCH), posteromedial condyle width (PMCW), and posterior condylar axis length (PCAL) dimension. They had comparatively smaller femur section aspect ratios (*p* < 0.005). They showed longer posterolateral condyle width (PLCW), anterior condyle mediolateral dimension (FRACML), anterolateral condyle height (ALCH), and femur resection anterior condylar mediolateral (FRACML) dimension (*p* < 0.005). They showed larger distal femur aspect ratio and resection aspect ratio (FAR, FRAR, *p* < 0.005). All selected prostheses showed ML undercoverage under similar AP dimensions, and ML undersizing of Attune systems was more obvious in three femoral prostheses.

**Conclusion:**

The distal femur morphological change of HA patient is shown as smaller AP dimension, narrow posterior condyle spacing, lower and shallower trochlear, thinner anterior condyle, wider and lower intercondylar notch and higher posterior‐lateral condyle. The selected prostheses showed ML undercoverage under similar AP dimensions. This typical morphological tendency of the distal femur seems to warrant consideration in the process of knee joint prosthesis upgrading.

## Graphical abstract

The immature knee joints of hemophilia patients are in a pathological environment for a long time during development, which leads to morphological variations of the mature knee joints.

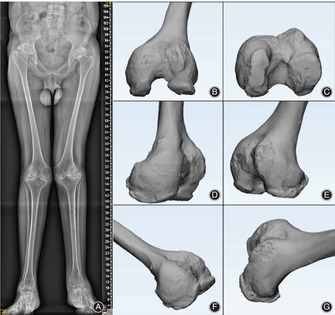

## Introduction

**FIGURE 1:**
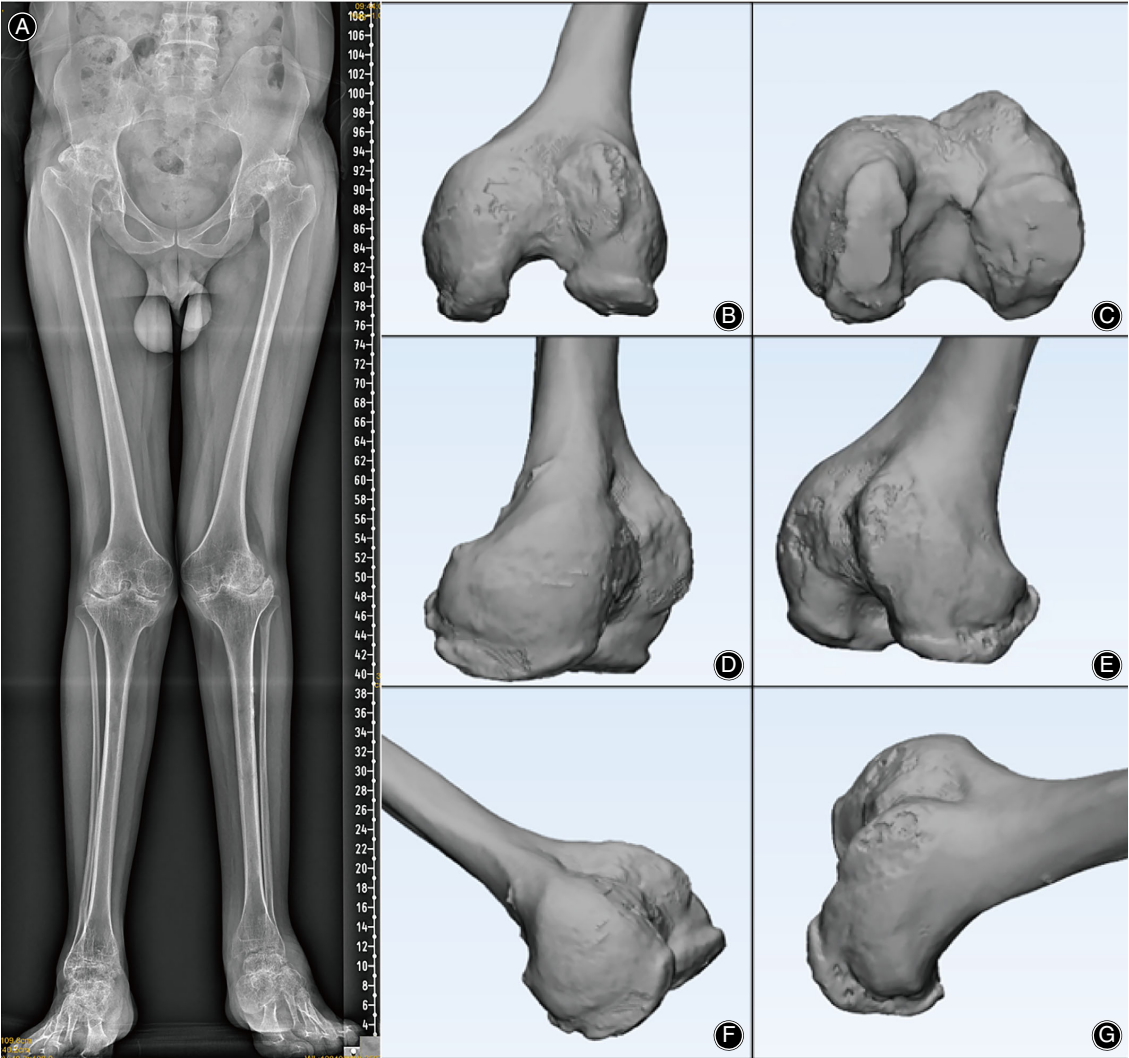
Radiological data of a patient with end‐stage HA. (A) Articular cavity loss, articular cartilage destruction and local developmental deformity are evident in the hip, knee, and ankle joints with end‐stage HA. (B–G) An apparent local developmental deformity of the distal femur for the mature knee joint with HA patient.

Hemophiliac arthritis (HA) is a rare, genetic, and destructive skeletal muscle hemorrhagic illness. Proliferative synovitis caused by repeated bleeding in the joint space is the most obvious pathological feature.^1^ The epiphyseal growth plate of immature joints shows abnormal development under the stimulation of this environment, and the large joints such as hip, knee, ankle, and elbow are often involved, among which the growth deformity of knee joint is the most obvious^2^ (Figure 1A). In radiology, there are obvious angular deformities, morphological or anatomical changes of knee joints in patients with end‐stage HA, such as metaphyseal hypertrophy, disappearance of joint space, subchondral bone cyst, erosion and collapse of articular surface, shortening of limbs, valgus deformity, proliferation of giant osteophytes, the lower and wider intercondylar notch, patellofemoral trochlear dysplasia etc.^3^, ^4^ (Figure 1B–G). This skeletal morphological change accompanied by the severe destruction of articular cartilage will seriously impact the knee joint function, and total knee arthroplasty (TKA) will have to be performed in the end‐stage.^5^

A good match between the prosthesis and the osteotomy surface is crucial for the recovery of knee function and physiological structure after surgery, which requires to mutually match in the design parameters of the selected prosthesis and the osteotomy surface. Currently, for the design of the prosthesis, most manufacturers refer to the knee joint morphology date of the osteoarthritis (OA) patients,^6^ in which the different variation ranges of the anteroposterior (AP) dimension, the mediolateral (ML) dimension, and aspect ratio of the osteotomy surface is used as the main reference information to design the different sizes.^7^, ^8^, ^9^ Although most of the prostheses on the market are designed based on it, more and more studies have found that the anatomical morphology of the knee joint is different according to different sides, races, genders, morphotype and bone survival state,^6^, ^10^, ^11^, ^12^, ^13^ so many types of prostheses have been developed. At present, the popular prostheses on the market have varying degrees of deficiencies on the matching degree of the osteotomy surface; even if the manufacturer provides various sizes for surgeons to choose from, the imperfect matching between the osteotomy surface and the prosthesis still faces many challenges.^14^

For HA patients who perform TKA, the abnormal development of knee joint will inevitably lead to morphological changes among which the development of distal femur is the most obvious,^15^ which brings great challenges to the matching and selection of femur prostheses during the TKA process. Although previous studies have shown that knee joint function has been effectively recovered in end‐stage HA patients after TKA, few studies quantitatively analyze the knee joint morphological parameters of HA patients and the prosthesis adaptability problems caused by knee joint morphological changes.

Therefore, this study aims to quantify the morphometrical parameters of HA patients’ knee joint with developing and maturing in pathological environment before and after simulated osteotomy, and compare these measurements with the dimensions of three currently commonly used femoral prostheses, and then clarify the clinical significance and developmental direction of knee joint morphology, so as to provide reference to update the prosthesis for prosthesis manufacturers.

## Material and Method

### Object

This study was approved by the ethics committee of our hospital (no. YX2022‐008‐F1) and all participants gave informed consent. The 50 male patients with end‐stage hemophilia knee arthritis registered and treated in our hospital from January 2018 to June 2022 were retrospectively selected to establish 3D bone model by the preoperative computed tomography (CT) data of the entire lower extremity.

Inclusion criteria: imaging of knee joint showed end‐stage degenerative arthritis; obvious pain symptoms for knee joint; failed conservative treatment; owning obvious operation indications. Exclusion criteria: the history of knee joint surgery, fracture, or rheumatoid arthritis; severe angle deformity for knee joint (more than 20°^16^); congenital dislocation of patella and maldevelopment; the history of knee bone fusion and the presence of knee tumor. The sample size of the object was estimated according to the experience of Ma et al.,^17^ that at least 50:37 subjects (male vs. female) with complete lower extremity data were included to conduct a comparative study for the prosthesis matching and morphological differences of knee joint in his study. Therefore, for the adequacy of comparison between groups, 50 male HA patients were randomly selected to be included in our study. Meanwhile, in order to conduct a multi‐group comparative study, we randomly selected the unilateral knee joint of 50 healthy male and 50 male OA patients who received TKA at the same time in our hospital's medical record system as the comparative group. The basic demographic data of the three groups such as age, height, BMI, side, the deformity angle of the lower limb are shown in Table 1.

**TABLE 1:** Basic data of three groups of patients.

		Healthy group (n = 50)	OA group (n = 50)	HA group (n = 50)	*p*
Age (years)		36.02 ± 7.37	**64.24 ± 5.30** ^**a**^	**34.38 ± 7.63** ^**c**^	<0.001
BMI (kg/m^2^)		23.42 ± 1.84	**23.97 ± 1.98** ^**a**^	**22.29 ± 2.49** ^**bc**^	<0.001
Height (cm)		172.62 ± 4.30	172.40 ± 3.52	173.10 ± 3.78	0.654
Side (Lt/Rt, n)		17/33	18/32	24/26	0.368
Deformity angle (°)	valgus	+0.85 ± 0.97 (39 knees)	**+4.1 ± 3.90** ^**a**^ **(22 knees)**	**+5.86 ± 4.4** ^**b**^ **(36 knees)**	<0.001
	varus	−2.00 ± 0.63 (11 knees)	−5.79 ± 3.11^**a**^ (28 knees)	−3.79 ± 2.29 (14 knees)	<0.001

*Note*: + indicates valgus (≥0°); − indicates varus (<0).

*Note*: X ^a,b,c^ indicates a statistical difference between the two groups (*p* < 0.05).

*Note*: X^a^: OA group vs. Healthy group; X^b^: HA group vs. Healthy group; X^c^: HA group vs. OA group.

### Acquisition of Lower Limb 3D Model Selection of Knee Prosthesis

The CT scanning range was unilateral whole lower limb with performing TKA belonging to HA or OA patients. The healthy group were selected from the healthy check‐up object who underwent lower limb CT examination in our hospital, and the cartilage had no degenerative changes. The whole lower limb was scanned using the spiral scanning mode of the LightSpeed 64 row volume CT machine (General Electric LightSpeed VCT, GE Healthcare, Milwaukee, WI, USA; image array 512 × 512, fastest time resolution 43 ms, contrast resolution 5 mm@ 0.3%, spatial resolution 15.42 lp/cm). During imaging, these objects remain supine with the entire lower limb straightened as much as possible. CT scanning of the entire ipsilateral lower limb was performed starting from the hip join. The slice thickness of each row of detectors is 0.625 mm. A three‐dimensional (3D) image of the entire lower limb was reconstructed using a 3D reconstruction computer program (Post‐processing 3D workstation, IntelliSpace Portal v6.0.5.02900; Philips Company, Germany). Finally, the original data scanned by the CT were imported into Mimics Medical 21.0 (Materialize, Leuven, Belgium) in DICOM format to reconstruct the 3D bone model of the entity lower extremity. In the Mimics software, the lower limb bone is extracted at first, and then the editable 3D model of the lower limb is established after the editing, segmenting, removing marginal osteophytes, smoothing, calculating steps of the bone model. Next, it is imported into the 3‐Matics software to simulate surgical cutting and measure the morphological parameters. Dimension parameters were recorded in millimeters (mm).

**FIGURE 2:**
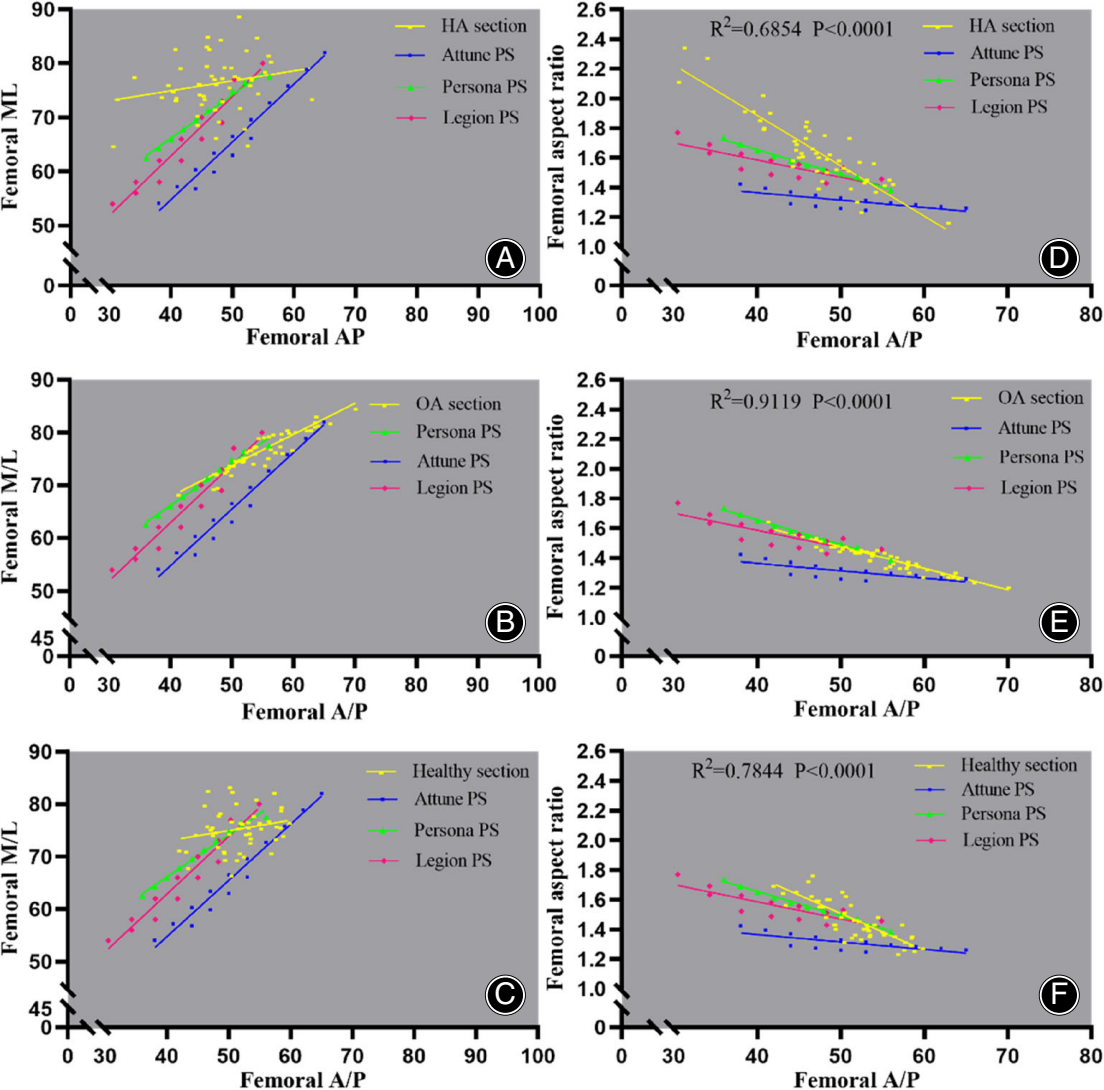
(A–C) The anterior‐posterior and the medial‐lateral dimensions of distal femur (M/L; A/P) after simulated osteotomy for 50 subjects in HA, OA, and healthy groups, respectively, showing close approximation of the Persona PS‐type prosthesis size to the morphologic data of OA group, while HA group were least close to the dimensions of the three groups of prostheses. (D–F) The femoral cutting aspect ratio versus the anterior‐posterior (A/P) measurements (mm) for HA, OA, and healthy group, respectively.

All the simulated osteotomy processes were performed according to the same surgical procedures, and three kinds of posterior‐stabilized (PS) prostheses with the same design characteristics were selected to compare the design parameters in Figure 2 (Attune PS Total Knee System: DePuy Synthes, Inc., Warsaw, IN, USA; Persona PS Total Knee System: Zimmer, Warsaw, IN, USA; Legion PS Total Knee System: Smith & Nephew, Memphis, TN, USA), which were highly recognized in clinical practice.

### Measurement Method

**FIGURE 3:**
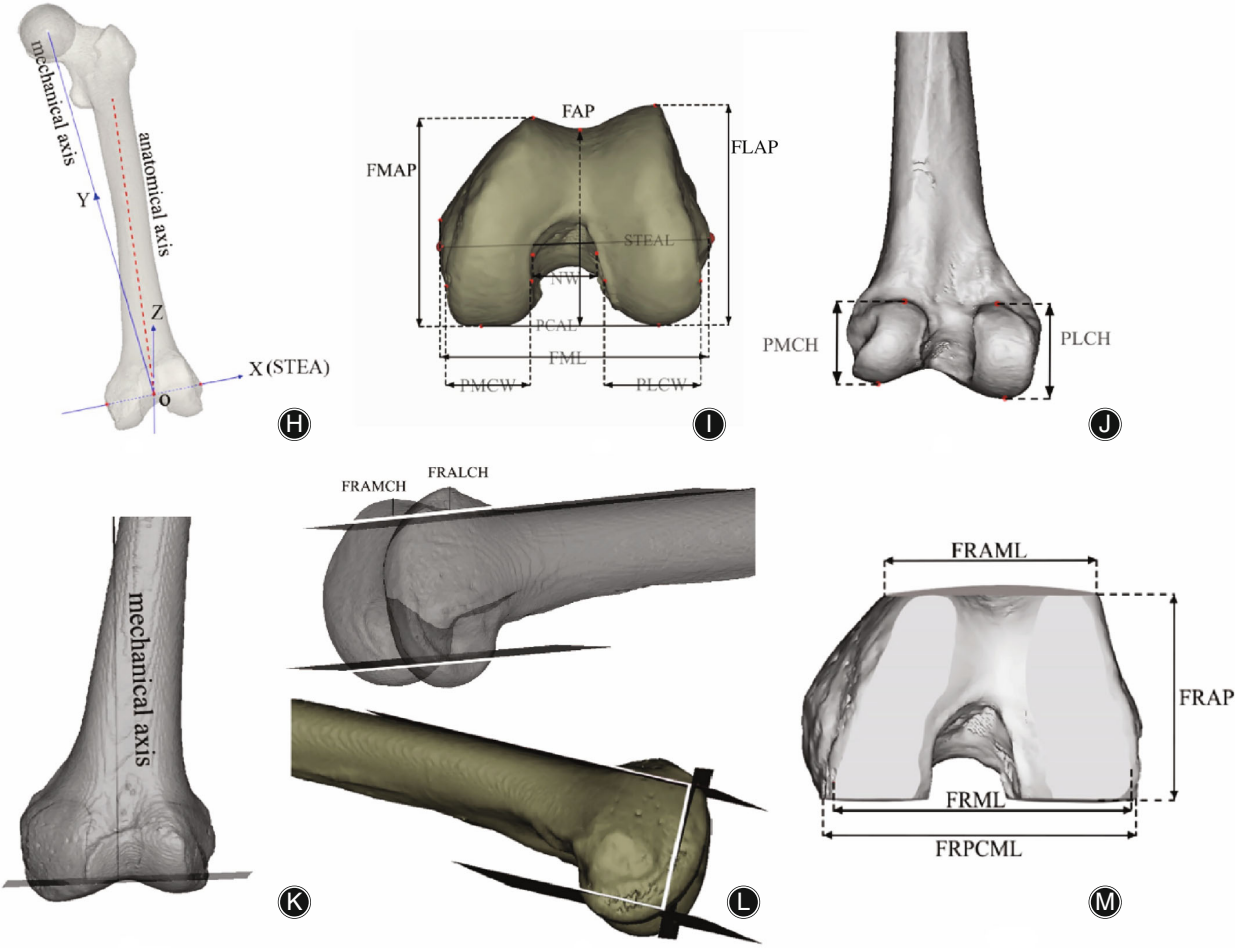
The process of morphological parameter measurement of the distal femur before and after simulated osteotomy. (H): A three‐dimensional coordinate system was established to simulate the process of distal femoral osteotomy. (I, J) Morphological parameters of distal femur before osteotomy. (K–M) The process of osteotomy and morphological parameters of osteotomy cutting for distal femur.

First, according to the generally accepted view, we determined the femur mechanical axis (FMA), anatomical axis (AA), surgical trans‐epicondylar axis (STEA), and three‐dimensional coordinate axis (3‐DCA) of simulated osteotomy^17^ (Figure 3). The midpoint of the STEA was defined as the center of the knee joint and the origin of the coordinate axis (O); the intersection of the mechanical axis and STEA defines the coronal plane; the axial plane is defined as the plane perpendicular to the coronal plane when passing through STEA; the plane passing the mechanical axis and perpendicular to the coronal plane is defined as the sagittal plane. The distal femoral valgus angle (DFVA) is defined as the angle between the anatomical and mechanical axes in the coronal plane, which determines the external rotation angle of the cutting plane.

The simulated cutting of the distal femur followed standardized operating procedures of TKA on the post‐reference‐type prosthesis. The simulated anterior condyle cut surface fitted to the cortex of the anterior edge of the distal femur and parallel to the plane projected STEA in the coronal plane.^17^ The posterior condyle simulated cut surface on the medial condyle was 10 mm, and lateral condyle was 8 mm from the lowest point on the medial and lateral posterior condyles, and parallel to the projected STEA in the coronal plane.^6^, ^18^ The distal osteotomy section was on the 9 mm above the lowest point of the medial condyle with rotating according to its own DFVA to obtain the osteotomy plane perpendicular to the mechanical axis.^19^

### Morphological Parameters before and after Surgical Simulation

The FAP, the femur mediolateral dimension (FML), the femur medial and lateral condyle anterior–posterior dimension (FMCAP, FLCAP), notch width (NW), the width and height of the posterolateral condyle width and height (PLCW, PLCH), the posteromedial condyle width and height (PMCW, PMCH), the posterior condylar axis length (PCAL), the STEA length (STEAL) (Figure 3I,J). Similarly, on the femur resection surface, the femur resection anteroposterior and mediolateral and posterior condyle mediolateral dimension (FRAP, FRML, FRPCML), the femur resection anteromedial and anterolateral condyle height (FRAMCH, FRALCH), the femur resection anterior condyle mediolateral dimension (FRACML) were recorded on the axial plane of the distal femur (Figure 3K–M). Aspect ratios before and after osteotomy (FAR, FRAR) were also recorded. In order to avoid data bias, all measurements were performed by three authors. The intra‐observer and inter‐observer reliability were determined by calculating the intra‐class correlation coefficient values of investigated parameters.

### Statistical Analysis

All data were statistically analyzed using SPSS 25.0 (IBM, Inc., Armonk, NY, USA). The count data were expressed with Mean ± SD.^−^χ test was used for counting data, and Bonferroni method was used for pairwise comparison. If the measurement data were consistent with normality and homogeneity of variance, one‐way ANOVA was used for statistical analysis, and the Bonferroni method was used for pairwise comparison. If not conforming to homogeneity of variance and normality, the multiple Kruskal–Wallis H test is used for statistical analysis, and the Bonferroni method of the multiple Kruskal–Wallis H test is used for pairwise comparison. Correlations between morphological data of the femur after the osteotomy and parameter of prosthesis were analyzed using linear correlation. *p* < 0.05 was considered the significant statistical difference.

## Result

### Morphological Comparison of Distal Femur before and after Simulated Osteotomy

#### The HA Group vs. the Healthy Group

Morphological parameters of distal femur such as FLAP, FMAP, FAP, NW, PLCH, PMCW, PCAL with before simulated cutting were significantly smaller in the HA group than healthy group, but LPCW, ALCH, FAR were significantly larger, and there were statistical differences between these parameters (*p* < 0.05). After simulated cutting, morphological parameters on the osteotomy surface such as FRAR, FRALCH, FRAMCH, FRACML were larger than healthy group, but FRAP of HA group was significantly smaller, and there were statistical differences between these parameters (*p* < 0.005). Compared with healthy group, the morphological deformity of the distal femur of HA patients was concentrated in the whole anterior–posterior dimension of the distal femur and NW, PLCH, PMCW, which development results showed as smaller overall, but PLCW development results showed as larger (Table 2).

**TABLE 2:** Comparison of morphology parameters for distal femur before and after osteotomy of three groups.

Morphological parameter	Healthy group (n = 50)	OA group (n = 50)	HA group (n = 50)	*p*
FLAP	65.55 ± 2.81	**63.75 ± 2.54** ^**a**^	**63.68 ± 4.97** ^**b**^	0.012
FMAP	62.74 ± 2.64	62.80 ± 2.92	**59.86 ± 6.99** ^**bc**^	0.024
FML	82.90 ± 3.02	83.28 ± 3.24	82.94 ± 5.29	0.993
FAP	59.37 ± 2.47	**57.29 ± 2.43** ^**a**^	**54.99 ± 5.4** ^**b**^	<0.001
FAR	1.40 ± 0.08	**1.46 ± 0.08** ^**a**^	**1.53 ± 0.19** ^**b**^	<0.001
NW	24.61 ± 2.48	24.60 ± 2.09	**21.05 ± 2.12** ^**bc**^	<0.001
PLCW	24.28 ± 2.55	**26.29 ± 3.26** ^**a**^	**26.71 ± 1.90** ^**b**^	<0.001
PLCH	39.54 ± 2.72	38.86 ± 4.48	**37.55 ± 1.77** ^**bc**^	<0.001
PMCW	26.07 ± 1.97	26.47 ± 1.44	**24.63 ± 4.16** ^**bc**^	0.021
PMCH	40.97 ± 2.29	40.56 ± 2.18	40.89 ± 3.53	0.506
PCAL	51.51 ± 3.31	**55.45 ± 5.83** ^**a**^	**49.33 ± 5.20** ^**bc**^	<0.001
STEAL	82.36 ± 3.05	82.02 ± 3.56	82.09 ± 4.98	0.684
FRAP	51.62 ± 4.44	**55.00 ± 5.73** ^**a**^	**47.10 ± 6.23** ^**bc**^	<0.001
FRML	75.27 ± 3.87	76.71 ± 3.65	76.25 ± 5.22	0.236
FRPCML	75.88 ± 3.73	76.40 ± 4.84	74.35 ± 4.72	0.060
FRAR	1.47 ± 0.13	**1.40 ± 0.08** ^**a**^	**1.65 ± 0.24** ^**bc**^	<0.001
FRALCH	8.29 ± 1.57	7.86 ± 2.60	**11.30 ± 3.14** ^**bc**^	<0.001
FRAMCH	4.15 ± 1.51	**5.13 ± 1.96** ^**a**^	**5.68 ± 3.50** ^**b**^	0.027
FRAML	50.50 ± 4.01	50.68 ± 5.48	**54.11 ± 5.85** ^**bc**^	0.001

*Note*: X ^a/b/c^ indicates a statistical difference between the two groups (*p* < 0.05).

*Note*: X^a^: OA group vs. Healthy group; X^b^: HA group vs. Healthy group; X^c^: HA group vs. OA group.

#### The HA Group vs. the OA Group

The FMAP, NW, PLCH, PMCW, and PCAL dimension with before simulated cutting were smaller in the HA group than OA group, and there were statistical differences between these parameters (*p* < 0.05). After simulated cutting, the FRAP of HA group was significantly smaller on the osteotomy surface but FRAR, FRALCH, and FRAML, which were significantly larger than OA group, showed statistical differences between these parameters (*p* < 0.05). Compared with OA group, morphological abnormalities of the distal femur for HA patients was concentrated in the smaller, shorter medial condyle anteroposterior dimension; narrower intercondylar fossa, posterior condylar space, and posteromedial condyle; and lower posterolateral condyle (Table 2).

The measurement results of morphological parameters exhibited excellent intra‐observer reliability with an intra‐observer correlation coefficient (ICC) > 0.75 (Table 3).

**TABLE 3:** Intra‐observer correlation coefficient (ICC) of measurements.

ICC	ICC intra‐group correlation coefficient	95% CI
FMCAP	0.907	0.872–0.933
FLCAP	0.906	0.870–0.932
FML	0.880	0.835–0.913
NW	0.926	0.897–0.946
PLCW	0.946	0.925–0.961
PLCH	0.908	0.873–0.933
PMCH	0.884	0.840–0.916
PMCW	0.884	0.840–0.916
PCAL	0.945	0.924–0.960
FRML	0.765	0.675–0.829
FAR	0.938	0.915–0.955
FRAR	0.896	0.856–0.924

#### Compared with Osteotomy Surface Size and Prosthesis Size

The resected surface ML width plotted against the AP length and prosthesis AP and ML sizes are shown in Figure 2. The Persona, Attune, and Legion systems had a smaller ML size than the total width of the resected of HA patients for a given femoral implant AP length, but only the best‐fit line of Persona systems was approximately closed to the best‐fit line of OA group date, and ML undersizing of Attune systems was more obvious in three groups. These implants tend to show under‐coverage the ML width of the resected distal femur of HA patients. FRAR of HA patients showed a higher ratio relative to the Persona, Attune, and Legion systems, but the best‐fit line of the FRAR and Persona and Legion systems was approximately closed in OA patients, as in the healthy group.

## Discussion

### Morphological Characteristics of Distal Femur

Our study compared the morphological development parameters of the distal femur between male HA, OA, and healthy groups, and showed that the FAP dimension of HA patients had a development tendency to be smaller than male healthy and OA people, but the development results were similar in the FML dimensions. Compared with the healthy group and OA group, the development characteristics of the distal femur in HA patients were mainly concentrated in the shorter dimension for FLAP, FMAP, FAP, PCAL, PMCW, NW, PLCH, FML/FAP; larger dimension for ALCH, AMCH, PLCW; and the similar dimension for FML, PMCH, STEAL.

Morphological parameters quantification of knee joint is helpful to understand the direction of the changes of anatomy development. Most of the previous studies focused on describing the differences in the morphological dimensions of knee joints in different genders, body types, races, heights, and age grades, while there were few reports on the morphological parameters of abnormal development of the knee joint in pathological states.^6^, ^10^, ^11^, ^12^, ^13^, ^20^ This study showed that the development characteristics of the distal femur relative to healthy and OA groups in HA patients were mainly concentrated in the shorter dimension for FLAP, FMAP, FAP, PCAL, PMCW, NW, PLCH, FML/FAP; larger dimension for ALCH, AMCH, PLCW; and the similar dimension for FML, PMCH, STEAL. A number of previous studies have suggested that morphology changes of the knee joint may be related to joint degeneration and the occurrence of end‐stage arthritis to a certain extent.^6^, ^21^, ^22^ In our study, it was only in the morphological results of the distal femur of HA and OA patients that the parameter range of changes was significantly smaller, which can definitely indicate that the abnormal changes of bone morphology is related to joint degeneration and bone destruction extent to some extent. However, the distal femur of OA patient also showed a few morphological changes.

Previous studies have suggested that uneven stress distribution in the lower limbs caused by secondary varus or valgus deformity of the HA knee for mature knees may be a factor in anatomical or morphological changes in the distal femur. However, more convincing studies believe that its root cause is synovitis caused by repeated intra‐articular hemorrhage in the knee cavity of immature HA patients and chronic stimulation of the epiphyseal growth plate by iron deposition of subchondral bone,^4^ which may impact the development trend of the epiphyseal growth plate in the mature stage of bone development, and then lead to local morphological or anatomical changes of the knee joint.^23^

### Morphological Characteristics of the Osteotomy Resection

Avoiding the prosthesis overhanging or undersized during TKA is an important procedure to prevent postoperative complications.^19^ In our study results, all prostheses showed a size mismatch and a difference in the aspect ratio at both larger and smaller sizes for HA group (Figure 2). In the HA group, when the AP size of prosthesis match well with the anteroposterior dimension of osteotomy surface, the undercovering probability of the prosthesis for the mediolateral dimension on the osteotomy surface is significantly higher than that of OA and healthy group. This phenomenon is the most obvious in the Attune‐type prosthesis, followed by the Legion‐type prosthesis, but the Persona‐type prosthesis has relatively better coverage. This indicates that a higher risk of exposure of cancellous bone on the HA osteotomy surface compared to the other two groups, which may be another source of increased postoperative knee bleeding in HA patients in addition to coagulation conditions.^24^, ^25^, ^26^, ^27^

On the osteotomy plane of the distal femur, the variation of FRML of HA, OA, and healthy subjects were positively correlated with FRAP to some extent, while FRAP and FRAR were negatively correlated, and the goodness of fit of the linear fit line of FRAR for HA was not as good as that of OA group and healthy group (R^2^ = 0.6854, *p* < 0.0001 vs. R^2^ = 0.9119 and R^2^ = 0.7844, *p* < 0.0001), suggesting that FAP also seems to influence FRAP after osteotomy to some extent, and this phenomenon is relatively more pronounced on OA resection. For healthy and OA group sections, the coverage extent of the ML of the prosthesis is similar when selected prostheses have similar AP dimension, which is similar to the conclusion of previous studies.^28^ For the HA section, the FRAR was significantly higher than OA and healthy group (1.65 ± 0.24 vs. 1.47 ± 0.13 and 1.40 ± 0.08), which indicated that when the prosthesis matching degree of the osteotomy surface of OA and healthy people was higher, the ML dimension increase of prosthesis could not adapt to the HA section. Such results portend the necessity for an appropriate increase in ML to fit the cut surface of HA in prostheses with similar AP dimension.

In terms of the Box AP‐ML fitted with the three prostheses, the prosthesis tended to be smaller in smaller FRAP sizes. Although the Attune‐ and Legion‐type prostheses offered multiple ML for AP size, the Attune‐type prosthesis showed a tendency to undercover on larger and smaller cutting surface (the determined AP and ML error range were within ±2.5 mm^29^), and the mismatch tendency of the Persona‐type prosthesis is relatively lower. For the aspect ratio, the fitting degree between the aspect ratio of osteotomy surface and the prosthesis was higher in OA and healthy group while the aspect ratio of HA section was significantly larger and more discrete. For different types of prosthesis, the aspect ratio of the Persona‐type prosthesis was the closest to that of the osteotomy surface, while the proximity of aspect ratio for Attune‐type prosthesis was lower. This result may suggest that the variation between the FRAP and the aspect ratio of the prosthesis is an important factor of mismatch.

The higher the mismatch rate between FRAP‐FRML and Box AP‐ML of prosthesis, the higher the possibility of postoperative complications. When the mismatch between the osteotomy surface and the prosthesis occurs, a compromise must be made to meet the prosthesis AP or ML. Therefore, if the prosthesis Box‐AP is fitted by osteotomy surface and gives up the ML, which will cause insufficient coverage of the prosthesis and exposes more cancellous bone, this may lead to increased intra‐articular postoperative bleeding, lasting pain, and osteolysis from wear debris and friction between the soft tissue and the osteotomy surface.^30^, ^31^ Similarly, when the ML of the osteotomy surface is fit and the AP is not, the amount of osteotomy of the anterior condyle or posterior condyle will be reduced, which may lead to change to osteotomy thickness of the anterior or posterior condyle, and eventually bring about joint stability destruction.^10^, ^32^, ^33^, ^34^, ^35^, ^36^ However, for these different types of prosthesis, although most surgeons will follow the principle of smaller than larger femoral prosthesis selection,^37^ the medium‐ and long‐term complications are still a noteworthy focus.

The quantification of morphological parameters to section surface is crucial for prosthesis renewal. From the results of our study, the insufficient coverage of the three prostheses is relatively common, which indicates that on the basis of this size, multiple alternative ML size prostheses can help to meet the osteotomy surface of morphological variation and achieve the purpose of increasing the chance of component matching.^19^ Therefore, it is necessary for manufacturers to explore this phenomenon and design a reliable femoral prosthesis for morphologically variable osteotomy surfaces to improve the long‐term outcome of TKA.

### Strengths and Limitations

This study not only enriches the information base of knee morphological parameters in people with special diseases, but also complements the morphological diversity of the distal femur in the Chinese population. Moreover, our research also has several limitations which deserve attention. First, our simulated osteotomy procedure was not performed separately according to the osteotomy method provided by different prosthesis manufacturers, which maybe produce angle differences on the osteotomy surface. Second, insufficient sample size and an imbalance of baseline data such as age, BIM, and deformity angle may also affect our results. However, insufficient sample size and imbalance of baseline data are unavoidable for case–control retrospective studies of this rare disease. In the future, we will cooperate with multiple research centers to explore this phenomenon and improve the database of related research on the disease. Finally, we only compared three post‐reference designs to prostheses, and whether our results would be applicable to other types of prostheses needs to be further explored. Nonetheless, the phenomenon of local morphological variation generated by abnormal development in pathological environment for HA patients should be taken seriously by both manufacturers and surgeons.

## Conclusion

Compared with healthy and OA group, the distal femur of HA patients shows shorter AP dimensions, narrower and lower intercondylar fossa and narrower posterior condyle distance, lower and shallower trochlear, thinner anterior condyle, higher posterior‐lateral condyle, but the mediolateral dimensions of the distal femur had no significant change. Moreover, the insufficient coverage of the HA osteotomy surface is common among the three popular types of prostheses when the AP dimensions of the prosthesis matched with the osteotomy surface, which is obvious for the Attune‐type prosthesis. Therefore, the characteristics and trends of development in the distal femur of HA patients appear to merit the attention of manufacturers during the knee prosthesis upgrading process.

## Conflict of Interest Statement

The authors declare that they have no conflicts of interest.

## Ethics Statement

Informed consent was obtained from all individual participants included in the study. Ethics Committee of The Second Hospital of Anhui Medical University approved this study (no. YX2022‐008(F1), approval date: 1‐Jan‐2022). The study was conducted according to the guidelines of the Declaration of Helsinki and its later amendments. All methods were carried out in accordance with relevant guidelines and regulations.

## Author Contributions

Yunfeng Yao developed the idea for the study. Ru Feng participated in its design and helped to draft the manuscript. Houlong Ye contributed to the acquisition and interpretation of data. Wang Fang contributed to the creation of the manuscript figures. Chun Zhang, Renfei Qi, Juehua Jing, and Yunfeng Yao revised the manuscript. All authors read and approved the final manuscript. Ru Feng, Wang Fang, and Houlong Ye contributed equally to this work.

## Data Availability

The datasets used and analyzed during the current study available from the corresponding author on reasonable request.
